# Prevalence, Persistence, and Factors Associated with SARS-CoV-2 IgG Seropositivity in a Large Cohort of Healthcare Workers in a Tertiary Care University Hospital in Northern Italy

**DOI:** 10.3390/v13061064

**Published:** 2021-06-03

**Authors:** Gitana Scozzari, Cristina Costa, Enrica Migliore, Maurizio Coggiola, Giovannino Ciccone, Luigi Savio, Antonio Scarmozzino, Enrico Pira, Paola Cassoni, Claudia Galassi, Rossana Cavallo, The Collaborative Group

**Affiliations:** 1 Hospital Medical Direction, Ospedale Molinette, University Hospital Città Della Salute e Della Scienza di Torino, 10126 Turin, Italy; gscozzari@cittadellasalute.to.it (G.S.); lsavio@cittadellasalute.to.it (L.S.); ascarmozzino@cittadellasalute.to.it (A.S.); 2 Microbiology and Virology Unit, University Hospital Città Della Salute e Della Scienza di Torino, 10126 Turin, Italy; rossana.cavallo@unito.it; 3 Clinical Epidemiology Unit, University Hospital Città Della Salute e Della Scienza di Torino, 10126 Turin, Italy; emigliore@cittadellasalute.to.it (E.M.); gciccone@cittadellasalute.to.it (G.C.); cgalassi@cittadellasalute.to.it (C.G.); 4 Cancer Epidemiology Unit, University Hospital Città Della Salute e Della Scienza di Torino, 10126 Turin, Italy; 5 Occupational Medicine Unit, University Hospital Città Della Salute e Della Scienza di Torino, 10126 Turin, Italy; mcoggiola@cittadellasalute.to.it (M.C.); enrico.pira@unito.it (E.P.); 6 Department of Medical Sciences, Pathology Unit, University of Turin, 10126 Turin, Italy; paola.cassoni@unito.it

**Keywords:** COVID-19 serological testing, SARS-CoV-2, health personnel, surveys and questionnaires, population surveillance

## Abstract

This observational study evaluated SARS-CoV-2 IgG seroprevalence and related clinical, demographic, and occupational factors among workers at the largest tertiary care University-Hospital of Northwestern Italy and the University of Turin after the first pandemic wave of March–April 2020. Overall, about 10,000 individuals were tested; seropositive subjects were retested after 5 months to evaluate antibodies waning. Among 8769 hospital workers, seroprevalence was 7.6%, without significant differences related to job profile; among 1185 University workers, 3.3%. Self-reporting of COVID-19 suspected symptoms was significantly associated with positivity (Odds Ratio (OR) 2.07, 95%CI: 1.76–2.44), although 27% of seropositive subjects reported no previous symptom. At multivariable analysis, contacts at work resulted in an increased risk of 69%, or 24% for working in a COVID ward; contacts in the household evidenced the highest risk, up to more than five-fold (OR 5.31, 95%CI: 4.12–6.85). Compared to never smokers, being active smokers was inversely associated with seroprevalence (OR 0.60, 95%CI: 0.48–0.76). After 5 months, 85% of previously positive subjects still tested positive. The frequency of SARS-COV-2 infection among Health Care Workers was comparable with that observed in surveys performed in Northern Italy and Europe after the first pandemic wave. This study confirms that infection frequently occurred as asymptomatic and underlines the importance of household exposure, seroprevalence (OR 0.60, 95%CI: 0.48–0.76).

## 1. Introduction

In December 2019, the novel β-coronavirus Severe Acute Respiratory Syndrome (SARS-CoV-2) was first described in Wuhan, China [1], and subsequently spread worldwide (https://covid19.who.int, accessed on 2 May 2021), with Northern Italy being one of the first areas affected outside China. In particular, the Piedmont region (Northwestern Italy, about 4.3 million inhabitants, half of them resident in the metropolitan area of Turin) currently accounts for more than 336,000 cases of coronavirus disease (COVID-19) and more than 11,000 deaths [2,3].

Serological testing can be useful with the main epidemiological purpose of estimating the magnitude of viral diffusion [4], especially because of the high frequency of asymptomatic/paucisymptomatic subjects and given the difficulties of an adequate contact tracing during a huge pandemic wave. Studies on serological response to SARS-CoV-2 have evidenced that nearly all patients (>95%) with laboratory-confirmed COVID-19 exhibit seroconversion [5,6], although with some peculiarities, especially in terms of occurrence and kinetic of antibody subtype IgM and individual variability [7,8,9,10,11,12]. Following the pandemic declaration, antibody assays for SARS-CoV-2 became rapidly available, the majority being developed for detection of immunoglobulin G (IgG) antibodies to the Spike (S) protein, although other antigens have been evaluated, including nucleocapsid protein, as well as pan-immunoglobulin assays which have also been developed (anti-S/RBD or anti-NCP) [13,14]. Both spike and nucleocapsid proteins are major immunogenic components of SARS-CoV-2, produced in abundant quantities 1–2 weeks after acute infection. Different platforms and assays have been developed, including enzyme-linked immunosorbent assay (ELISA), chemiluminescence (CLIA), bead-based flow cytometry, and lateral flow immunochromatography, with different features in terms of sensitivity and specificity [15,16,17,18].

Since the very beginning of the pandemic, Health Care Workers (HCW) have been identified as a subgroup at risk of developing the infection; furthermore, asymptomatic/paucisymptomatic HCW could represent a source of nosocomial outbreaks. Therefore, besides contact tracing activities and repeating PCR-RNA testing, several Health Care Institutions worldwide have promoted serological surveys as a measure of public health surveillance among HCW [19,20,21,22,23].

In May 2020, after the first pandemic wave, the Italian National Institute of Statistics (ISTAT) performed a seroprevalence survey on about 65,000 individuals from the general Italian population: among workers of the Health Care Sector, a national prevalence of 5.3% (95%CI 3.8–6.8%) was estimated, with large variations among areas, up to 9.8% (95%CI 6.5–13.1%) in areas of Northern Italy, where a higher rate of positivity was estimated in the general population [24].

In the present study, we primarily aimed at evaluating seroprevalence (SARS-CoV-2 IgG) among workers at the largest tertiary care University Hospital of Northwestern Italy located in Turin, and to examine clinical, demographic, and occupational factors associated with seropositivity. We also included in the study, workers of the University of Turin as a subgroup with no additional occupational risk a priori. Furthermore, the study aimed at retesting seropositive subjects after 5 months to evaluate antibodies waning, given the uncertainty of available data on IgG decline over time [4,7,23,25,26].

## 2. Materials and Methods

### 2.1. Study Design and Population

This is an observational, cross-sectional, and prospective study. All the workers at the University Hospital Città della Salute e della Scienza di Torino (CSS) (Turin, Piedmont region, Figure S1), including employees, students, medical residents, and fellows, for a total of 11,115 subjects, were invited to participate starting in April 2020. We also invited all the 3679 workers of the University of Turin (UNITO), an adult population with no professional risk for SARS-CoV-2 infection. Recruitment was on a voluntary basis, and the study was promoted by e-mail and through the CSS intranet web pages. Before blood sampling, subjects were asked to complete a questionnaire (largely comparable to the one used by ISTAT) to investigate demographic data, medical history, including previous COVID-19 symptoms, testing for SARS-CoV-2 by molecular assays, job profile, area of working, contacts with positive subjects at work or in the household. Seropositive subjects were immediately tested for SARS-CoV-2 RNA on a nasopharyngeal swab to exclude an acute infection if a recent swab (i.e., ≤12 days) was not available.

The first phase of the serosurvey for CSS workers started on 4 May 2020, at the end of the first pandemic wave (Figure S1); about 90% of subjects were tested by 12 June, with residual cases (and retesting of subjects with equivocal results) up to 31 July 2020. Among UNITO workers, the survey was conducted between 29 June and 29 July 2020.

All the subjects who tested positive or equivocal at the first phase were invited to be retested at the beginning of September 2020; blood samples were collected between 28 September and 20 November 2020, i.e., at the beginning of the second pandemic wave (Figure S1).

The study has been approved by the Local Ethics Committee (approval n. CS3/31, protocol n. 43078, 1 May 2020); each participating subject signed informed consent.

### 2.2. Serological Assay

Serological data on serum specimens were studied by the LIAISON^®^ SARS-CoV-2 S1/S2 IgG indirect chemiluminescent immunoassay (CLIA) (Diasorin, Saluggia, Italy), following the manufacturer’s instruction and using the LIAISON^®^ XL Analyzer. The assay uses specific recombinant S1 and S2 antigens coated on magnetic particles (solid phase) and mouse monoclonal antibodies to human IgG linked to an isoluminol derivative (conjugate). Antibody concentrations were calculated by the analyzer and expressed as arbitrary units (AU/mL), allowing for a qualitative grading of the results: <12.0 AU/m considered as negative; ≥15.0 AU/mL as positive. In the case of AU/mL between 12 and 15, the result was considered equivocal, and the assay was repeated at a 2-to-3 weeks interval [27].

### 2.3. Molecular Assay

SARS-CoV-2 RNA was studied in upper respiratory specimens (nasopharyngeal swab) by a commercial molecular test, Aptima^TM^ SARS-CoV-2 Assay with the Panther^TM^ Fusion System (Hologic, Italia, Rome), following the manufacturer’s instruction. This assay received Emergency Use Authorization by the Food and Drug Administration. Briefly, the assay combines the technologies of target capture, Transcription Mediated Amplification, and Dual Kinetic Assay and detects two conserved regions of the ORF1ab gene. Qualitative results were determined by a cut-off based on the total Relative Light Units and the kinetic curve type [28].

### 2.4. Statistical Analysis

Baseline characteristics of participants and seroprevalence data were summarized with absolute and relative (percentage) frequencies and reported as proportions with a 95% confidence interval (95%CI). Quantitative variables with non-parametric distribution (including IgG levels) were reported through medians (interquartile ranges, IQR). Differences between groups, for sociodemographic and anamnestic characteristics, were assessed through the Mann–Whitney U test for continuous variables and the chi-square test or Fisher’s exact test for categorical variables.

The association between SARS-CoV-2 antibody positivity and sociodemographic and occupational characteristics was evaluated using multivariable logistic regression models; Odds ratios (OR) and 95% confidence intervals (95%CI) were reported.

In the subgroup of subjects with positive or equivocal results in the first phase of the study, change in seropositivity, as well as variations in IgG level between the two phases were assessed; results are reported as absolute differences, also stratified by selected individual characteristics.

Statistical analyses were performed by Stata 15.1 software (StataCorp LP, College Station, TX, USA).

## 3. Results

Overall, 9954 individuals adhered to the study, including 8769/11,115 (78.9%) and 1185/3679 (32.2%) among CSS and UNITO personnel, respectively.

Table 1 reports the principal characteristics of CSS participants; median age was 49.2 years, most were females (73.5%) and about 87% Clinical Staff, including nurses (39%), physicians/surgeons (28.6%), and health care assistants (15%). Among participants from UNITO (Table S1), about 46% were Teaching and Research Staff and 54% Technical and Administrative Staff.

Seropositivity was detected in 7.6% (95%CI, 7.1–8.2%) and 3.3% (95%CI, 2.4–4.5%) in CSS and UNITO subjects, respectively (Table 1 and Table S1 report IgG positivity by demographic, clinical, and occupational features, and by type of previous contacts with persons with a diagnosis or suspected symptoms of COVID-19). Among positive or equivocal subjects, 450 underwent a nasopharyngeal swab, which proved negative in 441 cases and positive in 9, thus submitted to quarantine measures and clinical follow-up by the Occupational Medicine Unit. The results of the multivariable logistic regression are shown in Table 2: seropositivity was significantly associated with a history of previous COVID-19 contact, either at work or outside, with odds ratios ranging from 1.24 to 1.69 for work contacts (i.e., work in a COVID unit or other types of contacts, respectively) and up to 5.31 for household contacts (*p* < 0.001). A strong association with family contact was found in UNITO personnel as well (Table S2). Among CSS workers, being former smokers was associated with seropositivity (OR 1.36, 95%CI 1.09–1.69), while the opposite was observed for current smokers (OR 0.60, 95%CI 0.48–0.76) (Table 2); a similar pattern was also observed among UNITO workers, although with a larger imprecision of the estimates (Table S2). No further meaningful differences in relation to other features were found (Table 2 and Table S2).

Self-reporting of previous COVID-19 suspected symptoms or flu-like illness requiring a pharmacological treatment was associated with seropositivity (Table 3). Noteworthy, seroprevalence was 5.3% among individuals who reported no symptoms/signs suggestive for COVID-19 (i.e., 52% of enrolled subjects). This implies that approximately 27% reported no suspected signs/symptoms considering all seropositive CSS subjects.

Among CSS workers who tested positive, as expected, higher IgG levels were found in relation to reported previous COVID-19 suspected symptoms (or flu-like illness) and to positivity for SARS-CoV-2 RNA on a previous rhino pharyngeal swab; furthermore, higher levels were observed for household contacts and in older people, whereas lower levels were found among current smokers (Table 4).

**Figure 1:**
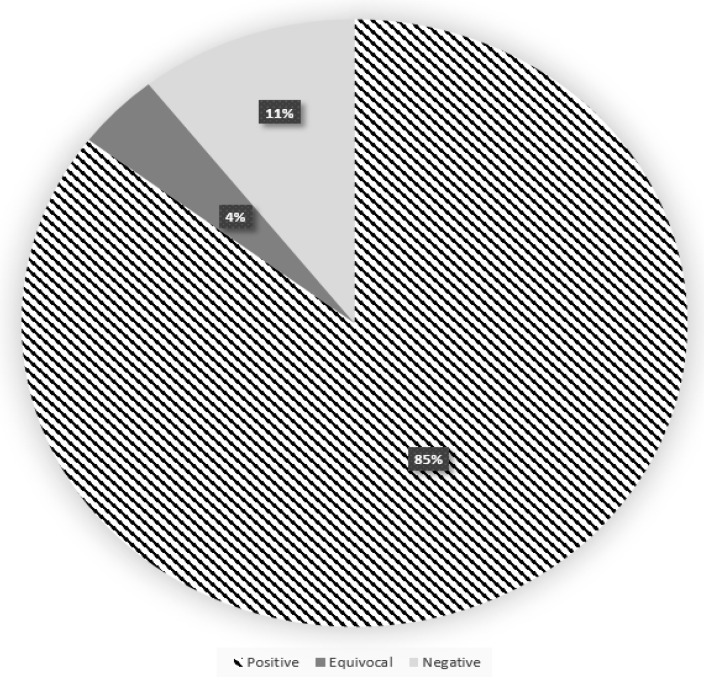
Results of the serological test repeated after 5 months among workers seropositive (≥15 AU/mL) at the first phase–CSS and UNITO workers.

**Figure 2:**
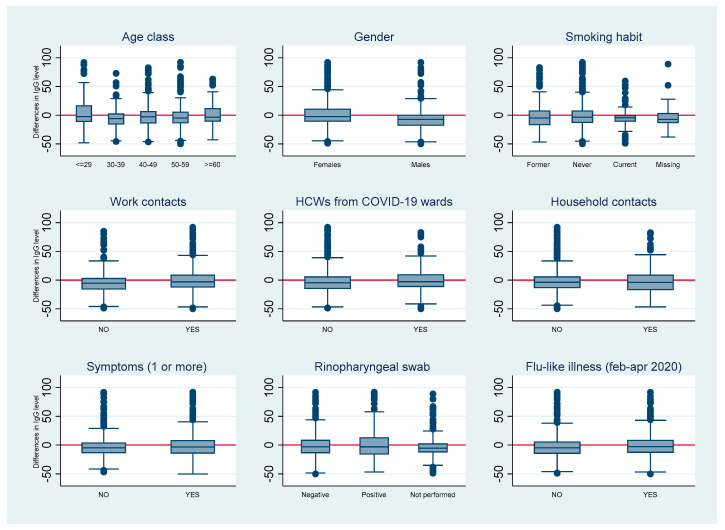
Absolute differences in IgG levels between 1st and 2nd phase among subjects who tested positive at the first phase, by some demographic, clinical, and occupational characteristics, CSS and UNITO workers.

Among individuals who tested positive or equivocal at the first phase of the study, 616/696 (88.5%) and 36/42 (85.7%) of CSS and UNITO, respectively, adhered to be retested. After a median of 5.2 months (IQR 4.66–5.75), 85.3% of previously positive subjects tested positive again, and 4.1% became equivocal; among those sero-reverted (10.6%), mostly (57/68) still had an antibody level between 3.80 e 11.99 AU/mL, whereas only 1.7% were below the threshold (<3.8) (Figure 1). Comparable results were found, limiting the analysis to CSS workers only (data not shown). We did not observe significant differences in the absolute change in IgG levels between the two time points (with an overall median decrease of about–5AU/mL) in relation to demographic, clinical, and occupational features, with the exception of a larger reduction among men (Figure 2).

## 4. Discussion

We here report the results of the largest seroprevalence study conducted until now in a Public Hospital in Italy, including about 8800 workers of a tertiary care Public University Hospital of Northwestern Italy, located in Turin. We estimated overall seropositivity of 7.6%, close to the 6.9% observed in a similar survey conducted among 5444 HCW of another Public Health Care Service of Turin (ASL Città di Torino), performed using the same serological assay, roughly in the same period of time [29]. By contrast, we found a reduced prevalence (3.3%) among 1185 workers of the University of Turin, with no professional risk of SARS-CoV2 exposure; noteworthy, this result is very close to the prevalence estimated by ISTAT for the general population of the Piedmont region (3%) [24], thus supporting the absence of additional occupational risk in our academic setting.

Comparison of seroprevalence data for HCW available in published literature is challenging [11,19,20,21,22,30], for several reasons.

At present, it is well established that seroprevalence among HCW largely mirrors the spread of the infection in the community served by the hospital [19,21,22,24,31,32,33]. In a large survey conducted in the Lombardy region (Northeastern Italy) after the first pandemic wave, the observed seroprevalence among HCW ranged widely, from 3% in the Varese area to 43% in the area of Bergamo, which were, respectively, the less and the most COVID19-affected Lombardy provinces during the first wave [33].

Timing of the serosurvey with respect to the pandemic wave is also a crucial aspect [19,20]; evidence regarding the kinetics of SARS-CoV-2 antibodies [4,7,11,23,34,35,36] suggests that antibody levels peak 2–4 weeks after infection, and then gradually decay and that levels are lower and reduce faster in mild/asymptomatic infections [9,10,11]. In our study, subjects who tested positive on May–June 2020 (after the first pandemic wave) showed lowered antibodies levels 5 months later (October–mid-November 2020), and about 10% of previously positive subjects sero-reverted in the same time frame, according to results from other studies [34,37]. In the second phase, we did not include a representative sample of HCW who tested negative at the first phase; however, among a selected group of 749 subjects who tested negative at the first survey, only 27 (3.6%) sero-converted by mid-November 2020, suggesting a moderate risk of infection (and reinfection) up to that date. Actually, the second phase of our survey was performed at the beginning of the second, larger, and long-lasting pandemic wave (Figure S1): it can be speculated that the second phase of the study would have produced different results if conducted one/two months later. Moreover, it should be noted that the waning effect of the serological response depends on the assay considered and the time post-infection, as previously reported [26], with positivity rate with the same serological assay used in the present study increasing up to 6–7 months, followed by a plateau phase or a decrease phase. In this regard, further informative data could also be obtained by evaluating levels of neutralizing antibodies, with studies having reported their longitudinal decline [38].

Further aspects that could hamper comparison of seroprevalence data are the use of serological assays with a different sensitivity [35], cut-off used for evaluating positivity that could impact on calculated specificity [39], different strategies for population selection and participation bias [20,40,41], availability and adequate use of personal protective equipment (PPE) [21], timing and type of local lockdown and quarantine measures. Not surprisingly, three meta-analyses of seroprevalence studies focused on HCW, reported a seroprevalence ranging from 0% to 45.3% [20,21,22]. It is reassuring that our prevalence is consistent with the meta-analytical estimates of the studies conducted in Europe by Galanis et al. (8.5%, 95%CI: 5.8–11.6%) [20] and by Hossain et al. (7.7%, 95%CI: 6.3–9.2%) [22], and with the overall prevalence found by Gomez et al. (7%, 95%CI: 4–11%) [21].

Seroprevalence surveys are primarily aimed at evaluating the “true” rate of infection in a population, allowing the capture of previous asymptomatic/paucisymptomatic infections. In our study, we found that about 27% of seropositive subjects did not report any previous symptom/illness, according to the ISTAT study on the general Italian population [24]. It is notable that our definition of a “symptomatic” subject was very sensitive, including reporting of at least one of 19 symptoms or flu-like illness. In the recent review performed by Alene et al. [42], not focused on HCW, the proportion of asymptomatic SARS-CoV-2 infections among 28 included studies ranged from 1.4% to 78.3%. Within the serosurveys performed in Northern Italy, the prevalence of asymptomatic subjects among HCW ranged from 11.9% [33] to 27.2% [31].

We investigated several demographics, clinical, and occupational factors which could be associated with a positive result at the serological test, using multivariable analysis in order to adjust for eventually unequal distribution of factors among different subgroups. Although male gender and advanced age represent well-known risk factors for the severity of COVID-19 disease [43], the present study did not find an increased risk of seropositivity according to age and gender. Similarly, as observed in other studies [33,44,45], we did not find an increased risk of seropositivity in subjects reporting at least one of the chronic disorders investigated. Furthermore, we did not report an association with flu-vaccine administration in the 2019–2020 season, as observed by others [33,46].

As already reported in several studies, we found a lower risk of seropositivity among active smokers [31,33,47,48], as well as lower levels of IgG among seropositive current smokers; conversely, we observed a higher risk of seropositivity among former smokers compared to never smokers. Some hypotheses have been put forward to explain the apparent lower incidence of SARS-COV-2 infection among active smokers [47,49,50], including a downregulation caused by nicotine on Interleukin-6 (IL-6), which plays a role in COVID-19 severity and interferes with the Angiotensin-Converting Enzyme 2, or an increased concentration of nitric oxide in the respiratory tract that could alter virus replication. However, until robust evidence from further investigations of the interaction between smoking and SARS-COV-2 infection becomes available, public health recommendations other than smoking banning cannot be proposed [49].

No significantly higher risk of seropositivity according to different professional HCW status was observed, similar to what was reported in another study [33], although other studies found higher seropositivity among nurses [21] or healthcare assistants [20,31]. As expected [20,21], our study evidenced that seroprevalence was associated with all types of reported contact, regardless of being with a suspect or confirmed symptomatic case of SARS-CoV-2. Compared to individuals who did not report the specific contact, those that occurred at work resulted in an increased risk of 69%, or 24% (in the case of working in a dedicated COVID ward). Instead, familiar contacts evidenced the highest risk, up to more than five-fold, and IgG levels were significantly higher for household contact as well. The relatively higher risk related to contacts in the household compared to those at work has been reported in other studies on HCW [20,21,31,33,45,51]. In addition, among UNITO personnel, a familiar contact was the main predictor of seropositivity, with a very high point estimate (OR = 11.8) but a large uncertainty (95%CI 4.8–29.5). We can speculate that the elevated risk of infection in the family context may be primarily related to the lack of use of PPE in the household; furthermore, also in the work environment, especially during the very first phase of the pandemic, the adequate use of PPE, especially in non-COVID dedicated wards, was less standardized [20]. In the study by Weinberger and colleagues on HCW in the first pandemic wave in a quaternary care hospital in Germany [52], unprotected private contact showed a trend to be associated with a higher rate of seroconversion (OR 3.38; 95%CI, 0.76–28.2, *p* = 0.08); similarly, unprotected risk contacts in the hospital either to an infected patient (OR 3.45; *p* = 0.03) or colleagues (OR 3.38; *p* = 0.04) had a statistically significant association with seroconversion.

The main strengths of the present study are the very large population included and the very high response rate among CSS workers (about 80%), supporting the representativeness of the HCW population examined and, therefore, a small risk of selection bias. Furthermore, questionnaires were filled in by subjects before blood sampling, thus strongly limiting a possible recall bias.

Further follow-up surveys of this cohort have been already scheduled in order to clarify better the crucial aspect of anti-SARS-CoV-2 IgG time curves and add data on SARS-CoV-2 immunological pathways.

This study shows a frequency of SARS-COV-2 infection among Health Care Workers comparable with that observed in surveys performed in Northern Italy and Europe after the first pandemic wave. It also confirms that infection frequently occurred asymptomatic and underlines the importance of exposure sources in the household.

## Figures and Tables

**Table 1:** Prevalence of seropositive by demographic, occupational, and clinical characteristics of study participants–CSS workers.

	All Participants	Seropositive
	*n* (%)	% (95%CI)
**Overall**	*n* = 8769	7.6 (7.1–8.2)
Age (median, IQR)	49.2 (39.1–56.0)	
**Age class**		
≤29 y	943 (10.8)	8.1 (6.4–10.0)
30–39 y	1376 (15.7)	7.3 (6.0–8.8)
40–49 y	2366 (27.0)	7.8 (6.8–9.0)
50–59 y	3098 (35.3)	7.5 (6.6–8.5)
≥60 y	986 (11.2)	7.5 (5.9–9.3)
**Gender**		
Female	6450 (73.5)	7.2 (6.5–7.8)
Male	2319 (26.5)	8.9 (7.8–10.1)
**Job profile**		
Clinical staff	7624 (86.9)	7.7 (7.1–8.3)
*Physician*	*2182 (28.6)*	*8.3 (7.2–9.6)*
*Biologist*	*145 (1.9)*	*10.3 (5.9–16.5)*
*Nurse*	*2981 (39.1)*	*7.5 (6.6–8.5)*
*Radiology Technician*	*192 (2.5)*	*6.8 (3.7–11.3)*
*Laboratory Technician*	*342 (4.5)*	*5.6 (3.4–8.5)*
*Obstetrician*	*146 (1.9)*	*8.9 (4.8–14.7)*
*Physiotherapist*	*111 (1.5)*	*4.5 (1.5–10.2)*
*Health care assistant (HCA)*	*1123 (14.7)*	*8.6 (7.1–10.4)*
*Other health care profiles*	*315 (4.1)*	*5.1 (2.9–8.1)*
*Not reported*	*87 (1.1)*	*6.9 (2.6–14.4)*
Administrative staff	807 (9.2)	7.3 (5.6–9.3)
IT/maintenance staff	325 (3.7)	6.2 (3.8–9.3)
*Not reported*	13 (0.2)	-
**Smoking habit**		
Never smokers	5230 (59.6)	7.8 (7.5–9.0)
Former smokers	1257 (14.3)	11.1 (9.5–13.0)
Current smokers	1973 (22.5)	5.0 (4.1–6.1)
*Not reported*	309 (3.5)	7.1 (4.5–10.6)
**BMI**		
Underweight (BMI < 18.5)	352(4.0)	7.1 (4.6–10.3)
Normal weight (BMI 18.5–25)	5236 (59.7)	7.4 (6.7–8.1)
Overweight (BMI 25–30)	2222 (25.3)	8.4 (7.3–9.6)
Obese (BMI > 30)	863 (9.8)	7.8 (6.1–9.8)
*Not reported*	*96 (1.1)*	*4.2 (1.1–10.3)*
At least one comorbidities *		
No	5058 (57.7)	8.1 (7.3–8.9)
Yes	3708 (42.3)	7.0 (6.2–7.9)
**Intake of drugs (regularly)**		
No	5058 (57.7)	8.0 (7.3–8.8)
Yes	3671 (41.8)	7.1 (6.3–8.0)
*Not reported*	*40 (0.5)*	*2.5 (0.6–13.2)*
**Flu vaccination (2019-20)**		
No	7162 (81.7)	7.6 (7.0–8.2)
Yes	1579 (18.0)	7.7 (6.5–9.2)
Not known	20 (0.2)	5.0 (0.1–24.9)
**Contacts at work**		
No	3870 (44.1)	5.4 (4.7–6.2)
Yes	4897 (55.8)	9.4 (8.6–10.2)
**Working in COVID-19 wards**		
No	6965 (79.3)	7.0 (6.4–7.6)
Yes	1802 (20.6)	10.1 (8.7–11.6)
*Not reported*	*2 (0.02)*	*-*
**Household contacts**		
No	8202 (93.5)	6.6 (6.1–7.2)
Yes	429 (4.9)	28.0 (23.8–32.5)
*Not reported*	*138 (1.6)*	*5.1 (2.1–10.2)*
**Other contacts**		
No	8528 (97.3)	7.4 (6.8–7.9)
Yes	234 (2.7)	17.1 (12.5–22.5)
*Not reported*	*7 (0.1)*	*14.3 (0.4–57.9)*

* Comorbidities: cardiovascular diseases, diabetes, allergic rhinitis, immune deficits, chronic respiratory diseases, renal diseases, hypertension, auto-immune diseases, neurological diseases, neoplasms.

**Table 2:** Multivariable logistic regression model (ORs and 95%CI) for predictors of seropositivity among workers of CSS.

	OR **	95%CI	*p*-Values
**Age**	1.00	0.99–1.01	0.388
**Gender**			
Female	1.00	REF	
Male	1.15	0.94–1.40	0.164
**BMI**			
Underweight (BMI < 18.5)	1.00	REF	
Normal weight (BMI18.5–25)	1.04	0.68–1.61	0.844
Overweight (BMI 25–30)	1.16	0.95–1.42	0.133
Obese (BMI > 30)	1.09	0.82–1.46	0.555
**Smoking habit**			
Never smokers	1.00	REF	
Former smokers	1.36	1.09–1.69	0.006
Current smokers	0.60	0.48–0.76	<0.001
Not defined	*0.99*	*0.60–1.62*	*0.959*
**At least one comorbidities**			
No	1.00	REF	
Yes	0.85	0.70–1.02	0.079
**Flu vaccination (2019-20)**			
No	1.00	REF	
Yes	0.95	0.76–1.19	0.638
**Intake of therapeutic drugs (regularly)**			
No	1.00	REF	
Yes	0.91	0.75–1.10	0.319
**Job profile:**			
Nurse	1.00	REF	
Administrative staff	1.30	0.94–1.81	0.112
IT/maintenance staff	1.00	0.61–1.66	0.987
Clinical staff (other than physician/nurse/HCA)	1.02	0.78–1.35	0.869
Physician	1.00	0.77–1.30	0.981
Health care assistant (HCA)	1.27	0.97–1.66	0.082
**Contacts at work**			
No	1.00	REF	
Yes	1.69	1.40–2.05	<0.001
**Working in COVID-19 wards**			
No	1.00	REF	
Yes	1.24	1.01–1.52	0.039
**Household contacts**			
No	1.00	REF	
Yes	5.31	4.12–6.85	<0.001
**Other contacts**			
No	1.00	REF	
Yes	1.28	0.86–1.92	0.224

** OR adjusted by all listed variables and also by type of employment contract and place of work.

**Table 3:** Associations between previous self-reported COVID-19 suspected symptoms or a flu-like illness between February–April 2020 and seropositivity-CSS workers.

	*n*	Seropositive % (95%CI)	OR *	95%CI	*p*-Values
At least one COVID symptom ** before blood sampling					
No	4576 (52.2)	5.3 (4.7–6.0)	1.00	REF	<0.0001
Yes	4193 (47.8)	10.1 (9.2–11.1)	2.08	(1.76–2.45)	
Flu-like illness between February and April 2020					
No	6727 (76.7)	5.6 (5.1–6.2)	1.00	REF	<0.0001
Yes	2033 (23.2)	14.1 (12.6–15.7)	2.82	(2.39–3.32)	

* All ORs adjusted by age and sex ** Previous self-reported COVID-19 suspected symptoms: temperature > 37. 5 °C, cough, sore throat, dyspnea, cold, asthenia, fatigue, hypo-ageusia, hypo-anosmia, headache/hemicrania, diarrhea, nausea/vomiting, decreased appetite, abdominal pain, muscle pain, general malaise, confusion, conjunctivitis, skin rash/skin symptoms.

**Table 4:** Distribution of IgG levels at the first survey by demographic, clinical, and occupational characteristics among seropositive (≥15 AU/mL) CSS workers.

		IgG Level in Seropositive (*n* = 668)	
	*n*	Median (Q1, Q3)	*p*-Values *
**Age class**			
≤29 y	76	31.4 (22.3, 49.5)	0.048
30–39 y	101	39.5 (23.9, 58.3)	
40–49 y	185	37.7 (24.9, 61.5)	
50–59 y	232	44.4 (23.6, 84.5)	
≥60 y	74	55.0 (22.8, 102.0)	
**Gender**			
Female	462	39.3 (24.0, 72.3)	0.850
Male	206	41.6 (22.7, 82.0)	
**Smoking habit**			
Never smokers	140	48.0 (25.6, 93.1)	<0.001
Former smokers	407	41.4 (24.2, 75.0)	
Current smokers	99	27.1 (18.8, 47.9)	
Not reported	22	37.3 (29.2, 61.0)	
**Job profile:**			
Administrative staff	59	29.6 (22.5, 60.9)	0.330
Clinical staff	589	40.3 (24.0, 77.5)	
IT/maintenance staff	20	50.9 (22.8, 75.4)	
**Working contacts**			
No	210	41.0 (22.4, 71.5)	0.675
Yes	458	39.7 (24.1, 78.9)	
**Working in COVID-19 wards**			
No	486	41.7 (23.6, 82.0)	0.060
Yes	182	35.6 (23.8, 58.3)	
**Household contacts**			
No	541	37.5 (22.8, 72.3)	0.004
Yes	120	46.8 (30.9, 85.8)	
**At least one symptom suspected for COVID-19 (prior to blood draw)**			
No	243	32.0 (22.5, 62.2)	<0.001
Yes	425	44.1 (25.8, 82.3)	
**Rhino pharyngeal swab for SARS-CoV-2 RNA**			
Negative	277	38.6 (24.0, 69.2)	<0.001
Positive	180	49.5 (32.8, 92.9)	
Not performed	211	32.0 (20.6, 58.3)	
**Flu-like illness between February–April 2020**			
No	380	32.5 (21.9, 61.0)	<0.001
Yes	287	49.2 (30.9, 91.9)	

* Mann–Whitney test.

## Data Availability

The data presented in this study are available within the article and supplementary material.

## Supplementary Materials

The following are available online at https://www.mdpi.com/article/10.3390/v13061064/s1, Table S1: Prevalence of seropositive by demographic and occupational characteristics and by type of contact with persons with a diagnosis or suspected symptoms of COVID-19 among UNITO workers. Table S2: Multivariable logistic regression model (ORs and 95%CI) for predictors of seropositivity among workers of UNITO; Figure S1: Number of daily access (emergency room visits and/or hospitalizations) to CSS city of Turin, of COVID-19 patients during 2020. Vertical lines identify the periods of the two seroprevalence surveys on CSS workers.
