# Supplementary material for: Prevalence, Persistence, and Factors Associated with SARS-CoV-2 IgG Seropositivity in a Large Cohort of Healthcare Workers in a Tertiary Care University Hospital in Northern Italy
Source: Viruses. 2021 Jun 3;13(6):1064. doi: 10.3390/v13061064 (PMC8229066; doi:10.3390/v13061064)
Supplement: Supplementary file 1 [file viruses-13-01064-s001.zip › viruses-1230032-supplementary.pdf]

viruses

MDPI

Table S1. – Prevalence of seropositive by demographic and occupational characteristics and by type of contact with persons with a diagnosis or suspected symptoms of COVID-19 among UNITO workers.

	**All participants *N* (%)**	**Seropositive % (95%CI)**
Overall	*N*=1185	3.3 (2.4-4.5)
Age (median, IQR)	50.5 (44.1-56.5)	
**Age class**		
≤29 yrs	10 (0.8)	-
30-39 yrs	146 (12.3)	1.4 (0.2-4.9)
40-49 yrs	413 (34.9)	3.6 (2.0-5.9)
50-59 yrs	446 (37.6)	2.9 (1.6-4.9)
≥ 60 yrs	170 (14.4)	5.3 (2.4-9.8)
**Gender**		
Female	757 (63.9)	3.3 (2.2-4.8)
Male	428 (36.1)	3.3 (1.8-5.4)
**Job profile:**		
Teaching and Research Staff	544 (45.9)	2.6 (1.4-4.3)
Technical and Administrative Staff	641 (54.1)	3.9 (2.5-5.7)
**Smoking habit**		
Never smokers	842 (71.1)	3.1 (2.0-4.5)
Former smokers	95 (8.0)	4.2 (1.2-10.4)
Current smokers	146 (12.3)	4.1 (1.5-8.7)
Not reported	*102 (8.6)*	*2.9 (0.6-8.4)*
**BMI**		
Underweight (BMI <18.5)	44 (3.7)	2.3 (0.1-12.0)
Normal weight (BMI18.5-25)	728 (61.4)	3.6 (2.3-5.2)
Overweight (BMI 25-30)	298 (25.2)	2.7 (1.2-5.2)
Obese (BMI >30)	95 (8.0)	3.2 (0.7-8.9)
Not reported	*20 (1.7)*	*5.0 (0.1-24.9)*
**Contacts at work**		
No	1151 (97.1)	3.2 (2.3-4.4)
Yes	33(2.7)	6.1 (0.7-20.2)
Not reported	*1 (0.1)*	-
**Household contacts**		
No	1139 (96.1)	2.6 (1.8-3.7)
Yes	45 (3.8)	20.0 (9.6-34.6)
Not reported	*1 (0.1)*	-
**Other contacts**		
No	1153 (97.3)	3.4 (2.4-4.6)
Yes	31 (2.6)	-
Not reported	*1 (0.1)*	-

Table S2. Multivariable logistic regression model (ORs and 95% CI) for predictors of seropositivity among workers of UNITO.

	**OR**	**95%CI**	**p-values**
**Age**	**1.03**	**0.98-1.07**	**0.208**
**Gender**			
Female	1.00		
Male	1.19	0.56-2.54	0.652
**BMI**			
Underweight (BMI <18.5)	1.00		
Normal weight (BMI18.5-25)	0.86	0.11-6.76	0.884
Overweight (BMI 25-30)	0.64	0.27-1.52	0.313
Obese (BMI >30)	0.57	0.13-2.58	0.465
**Smoking habit**			
Never smokers	1.00		
Former smokers	1.22	0.40-3.79	0.727
Current smokers	0.88	0.32-2.45	0.810
Not reported	0.55	0.12-2.49	0.441
**At least one comorbidities***			
No	1.00		
Yes	1.87	0.85-4.15	0.122
**Flu vaccination (2019-20)**			

No	1.00		
Yes	0.70	0.24-2.04	0.515
**Intake of therapeutic drugs (regularly)**			
No	1.00		
Yes	1.22	0.54-2.76	0.637
**Job profile:**			
Teaching and Research Staff	1.00		
Technical and Administrative Staff	1.60	0.77-3.34	0.209
**Contacts at work**			
No	1.00		
Yes	1.24	0.23-6.71	0.799
**Household contacts**			
No	1.00		
Yes	11.89	4.79-29.46	<0.001

*Comorbidities: cardiovascular diseases, diabetes, allergic rhinitis, immune deficits, chronic respiratory diseases, renal diseases, hypertension, auto-immune diseases, neurological diseases, neoplasms.

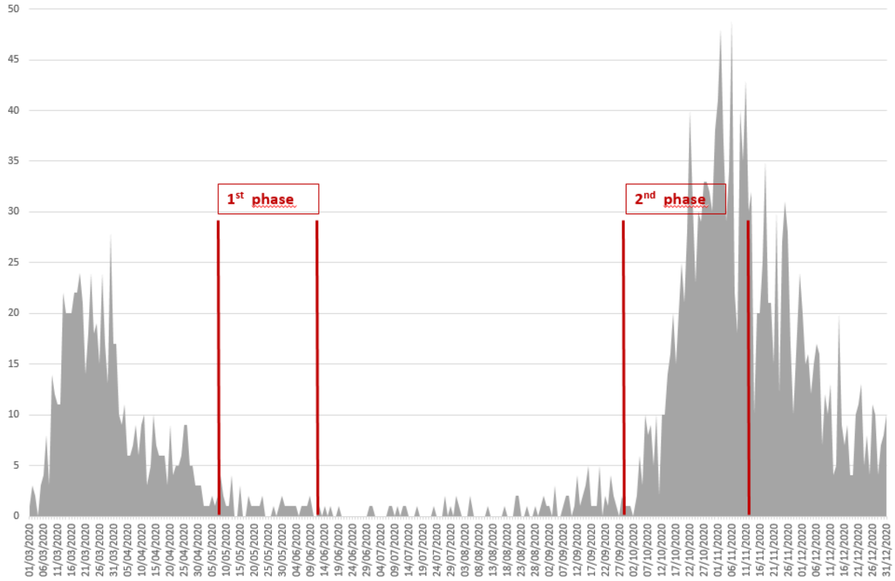

Figure S1. Number of daily access (emergency room visits and/or hospitalizations) to CSS – city of Turin, of COVID-19 patients during 2020. Vertical lines identify the periods of the two seroprevalence surveys on CSS workers.
